# Significance testing and genomic inflation factor using high‐density genotypes or whole‐genome sequence data

**DOI:** 10.1111/jbg.12419

**Published:** 2019-06-19

**Authors:** Sanne van den Berg, Jérémie Vandenplas, Fred A. van Eeuwijk, Marcos S. Lopes, Roel F. Veerkamp

**Affiliations:** ^1^ Animal Breeding and Genomics Wageningen University and Research Wageningen The Netherlands; ^2^ Biometris Wageningen University and Research Wageningen The Netherlands; ^3^ Topigs Norsvin Research Center Beuningen the Netherlands

**Keywords:** DNA analysis, genome‐wide association studies, pig population, significance testing, whole‐genome sequence

## Abstract

Significance testing for genome‐wide association study (GWAS) with increasing SNP density up to whole‐genome sequence data (WGS) is not straightforward, because of strong LD between SNP and population stratification. Therefore, the objective of this study was to investigate genomic control and different significance testing procedures using data from a commercial pig breeding scheme. A GWAS was performed in GCTA with data of 4,964 Large White pigs using medium density, high density or imputed whole‐genome sequence data, fitting a genomic relationship matrix based on a leave‐one–chromosome‐out approach to account for population structure. Subsequently, genomic inflation factors were assessed on whole‐genome level and the chromosome level. To establish a significance threshold, permutation testing, Bonferroni corrections using either the total number of SNPs or the number of independent chromosome fragments, and false discovery rates (FDR) using either the Benjamini–Hochberg procedure or the Benjamini and Yekutieli procedure were evaluated. We found that genomic inflation factors did not differ between different density genotypes but do differ between chromosomes. Also, the leave‐one‐chromosome‐out approach for GWAS or using the pedigree relationships did not account appropriately for population stratification and gave strong genomic inflation. Regarding different procedures for significance testing, when the aim is to find QTL regions that are associated with a trait of interest, we recommend applying the FDR following the Benjamini and Yekutieli approach to establish a significance threshold that is adjusted for multiple testing. When the aim is to pinpoint a specific mutation, the more conservative Bonferroni correction based on the total number of SNPs is more appropriate, till an appropriate method is established to adjust for the number of independent tests.

## 1 INTRODUCTION

Genome‐wide association studies (GWAS) aim to associate single nucleotide polymorphism (SNP) with a trait of interest in order to get a better understanding of the genetic architecture and to improve the accuracy and persistency of genomic prediction (VanRaden, Tooker, O'Connell, Cole, & Bickhart, 2017). A SNP is classified as associated SNP when it exceeds a significance threshold usually expressed as the –log10 (*p*‐value). These associated SNPs could however also be a false positive, leading to wrong conclusions about the genetic architecture underlying a trait or to a suboptimal subset of SNPs for genomic prediction. With the increasing number of SNPs used for GWAS, especially up to imputed whole‐genome sequences (iWGS), the number of false‐positive associations is expected to increase and choosing an appropriate significance threshold becomes an issue. Although the whole concept of significance thresholds should not be misused to assume causality and reproducibility of SNP effects (Baker, 2016; Wasserstein, Schirm, & Lazar, 2019), it is a useful concept for animal breeding to preselect and differentially weight SNPs in across‐breed genomic prediction (Raymond et al., 2018).

One approach to reduce the number of false positives is genomic control based on the genomic inflation factor. The genomic inflation factor expresses the deviation of the distribution of the observed test statistic compared to the distribution of the expected test statistic. High genomic inflation factors are caused by population stratification, strong linkage disequilibrium (LD) between SNPs, strong association between SNPs and phenotypes, and systematic bias (Devlin & Roeder, 1999; Hinrichs, Larkin, & Suarez, 2009; Reich & Goldstein, 2001; Zheng, Freidlin, & Gastwirth, 2006). Within livestock populations, we deal with genotype data that are imputed to higher density SNP data or even to whole‐genome sequence data, and also with individuals that are closely related to each other. Henceforth, the question is how the genomic inflation factor is affected in a GWAS with such data. Furthermore, different LD patterns across chromosomes may exist (Veroneze et al., 2013), and therefore, we would also expect different genomic inflation factors across chromosomes. To our knowledge, this has not been investigated yet.

In addition to applying genomic control, choosing an appropriate significance threshold can also control the number of false positives. A significance threshold of −log10 (*p*‐value) >7.2 (Welter et al., 2013) is commonly accepted in human genetic studies, while there is no commonly agreed threshold for livestock studies, such as for a pig breeding population. Permutation testing is a method to derive a empirical significance threshold that accounts for multiple testing and allows for the statistical dependence between SNPs (Churchill & Doerge, 1994). However, permutation testing is computationally intensive, especially when a large number of SNPs are involved in a large population.

Less computationally intensive approaches to account for multiple testing, which could cause an higher number of false positives, involve adjusting the significance threshold for either the family‐wise type 1 error rate or the false discovery rate (FDR). The family‐wise type 1 error rate aims to minimize the probability of finding at least one false positive. The family‐wise type 1 error rate is usually controlled by applying a Bonferroni correction that adjusts the significance threshold by the number of independent tests. In livestock, the number of independent tests is usually defined as the total number of SNPs or as the number of independent chromosome fragments, defined as regions of the genome that explain unique genetic variation (Duggal, Gillanders, Holmes, & Bailey‐Wilson, 2008; Ricard et al., 2017). Using the total number of SNPs can result in too conservative thresholds because it violates the assumption of independency between tests (Duggal et al., 2008; Nicodemus, Liu, Chase, Tsai, & Fallin, 2005). In contrast to the Bonferroni correction, the FDR does not aim to control the probability of finding at least one false positive but allows a proportion of the positive results to be false. Two commonly used FDR adjustments, among others, are the Benjamini and Hochberg (Benjamini & Hochberg, 1995) approach and the Benjamini and Yekutieli approach (Benjamini & Yekutieli, 2001).

In published studies in pig populations, threshold values for −log10 (*p*‐value) ranged from 3.3 to 6, using either no multiple testing correction, a Bonferroni correction, the false discovery rate, or genomic control (Do et al., 2013; Hao et al., 2017; Le, Christensen, Nielsen, & Sahana, 2017; Ma et al., 2013; Sahana, Kadlecová, Hornshøj, Nielsen, & Christensen, 2013; Sanchez et al., 2014; van Son et al., 2017). Therefore, the objective of this study was to investigate genomic control and different significance testing procedures using data from a commercial pig breeding scheme to establish guidelines for significance testing in a GWAS using either medium‐density genotypes, high‐density genotypes or imputed WGS (iWGS).

## 2 MATERIALS AND METHODS

### 2.1 Data

The data set for a Large White (LW) line was provided by Topigs Norsvin. The data set included precorrected phenotypes for number of teats of 4,964 Large White (LW line) pigs (Lopes et al., 2017), medium‐density genotypes (34,588 SNPs) and high‐density genotypes (491,169 SNPs). High‐density genotypes were imputed to WGS with Beagle 4.0 (Browning & Browning, 2009), using a multi‐line reference population of 168 animals of which 32 individuals originate from the LW line (van den Berg et al., 2019). After imputation and quality control, 10,212,687 SNPs that had a Beagle imputation accuracy (*R* ^2^) > 0.6 were considered for iWGS and the average Beagle *R* ^2^ was 0.93.

### 2.2 Single‐SNP genome‐wide association study

A single‐SNP GWAS was performed for medium density, high density and imputed WGS (iWGS), applying a mixed linear association model with a leave‐one‐chromosome‐out (LOCO) approach as implemented in GCTA version 1.25.2 (Yang, Manolio, et al., 2011; Yang, Zaitlen, Goddard, Visscher, & Price, 2014). The model was as follows:

(1) y=1μ+xb+u+e

where **y** is a vector of the phenotypes, µ is the mean, b is the fixed effect of the SNP tested for association, **x** is a vector of the SNP genotypes coded as 0, 1 or 2, **u** is a vector of random polygenetic effect and **e** is a vector of residuals. The residuals were distributed following a normal distribution *N*(**0**,**I**σ*_e_* ^2^) with σ*_e_* ^2 ^being the residual variance. The random polygenetic effect followed a normal distribution **u** ~ *N*(**0**,**G**σ*_g_* ^2^), where **G** is the genomic relationship matrix (Yang, Manolio, et al., 2011) for which the chromosome of the SNP tested is ignored and σ*_g_* ^2^ is the genetic variance.

### 2.3 Genomic control

The chi‐square test statistics, needed for the computation of the genomic inflation factors, were calculated from the *p*‐values assuming 1 degree of freedom. The genomic inflation factor was defined as the median of the observed chi‐squared test statistics divided by the expected median of the corresponding chi‐squared distribution and was computed for each chromosome separately and for the whole genome for the different densities.

### 2.4 Definition of the significance threshold

Three approaches to establish significance thresholds were evaluated: (a) permutation testing, (b) the Bonferroni correction and (c) the FDR. The definition of the significance threshold was expressed as the –log10 (*p*‐value). The established significance thresholds were compared between medium‐density, high‐density and iWGS genotypes.

The permutation test followed the procedure for the estimation of the experimental critical values that was proposed by Churchill and Doerge (1994). The phenotypes were randomly shuffled and subsequently used for a single‐SNP GWAS analysis. A total of 1,000 permutations were performed, and the maximum –log10 (*p*‐value) of each permutation was recorded. The –log10 (*p*‐value) significance threshold was defined as the 95th percentile of the ordered recorded values. Using either medium‐density genotypes, high‐density genotypes or iWGS, the permutation test was performed for only 3 chromosomes to limit the computational costs. Chromosomes 4, 7 and 10 were chosen to represent different levels of association.

The Bonferroni correction divides the probability of having at least one false‐positive result when the null hypothesis (*H*_0_) is true (*α*) by the number of independent tests. In this study, *α* was set to 0.05. The number of independent tests was either the total number of SNPs or the number of independent chromosome fragments. The number of independent chromosome fragments (*M*_e_) was calculated as follows (Goddard, Hayes, & Meuwissen, 2011):

(2) $$M_{e}=\frac{1}{Var\left(G_{\mathrm{ij}}\right)}$$

where **G** is the genomic relationship matrix computed following the first method of VanRaden (2008) with SNPs of all chromosomes. Goddard et al. (2011) showed that this estimation of *M*_e_ is equal to the inverse of the average LD (measured as *r* ^2^) across the whole genome.

Thresholds adjusted for the FDR were established using two different procedures. The first procedure evaluated was a three‐step strategy proposed by Benjamini and Hochberg (BH) (Benjamini & Hochberg, 1995). First, the *p*‐values were ordered in ascending order. Second, *k* was determined as the rank of *max* $\left\{p_{\left(i\right)}<i\frac{\alpha }{m}\right\}$, with *α* = 0.05, and *m* being the total number of SNPs. Third, the *p*‐value at rank *k* was defined as the threshold and all SNPs with a rank smaller than *k* are declared significant. The second procedure evaluated was proposed by Benjamini and Yekutieli (BY) (Benjamini & Yekutieli, 2001). This procedure followed the same steps as the BH one, except that *k* was defined as the rank of max$\left\{p_{\left(texti\right)}<i\frac{\alpha }{m*\sum_{i=i}^{m}\frac{1}{i}}\right\}$. Both false discovery rates were estimated using the R package “mutoss” (Blanchard et al., 2010).

## 3 RESULTS

### 3.1 GWAS

**Figure 1:**
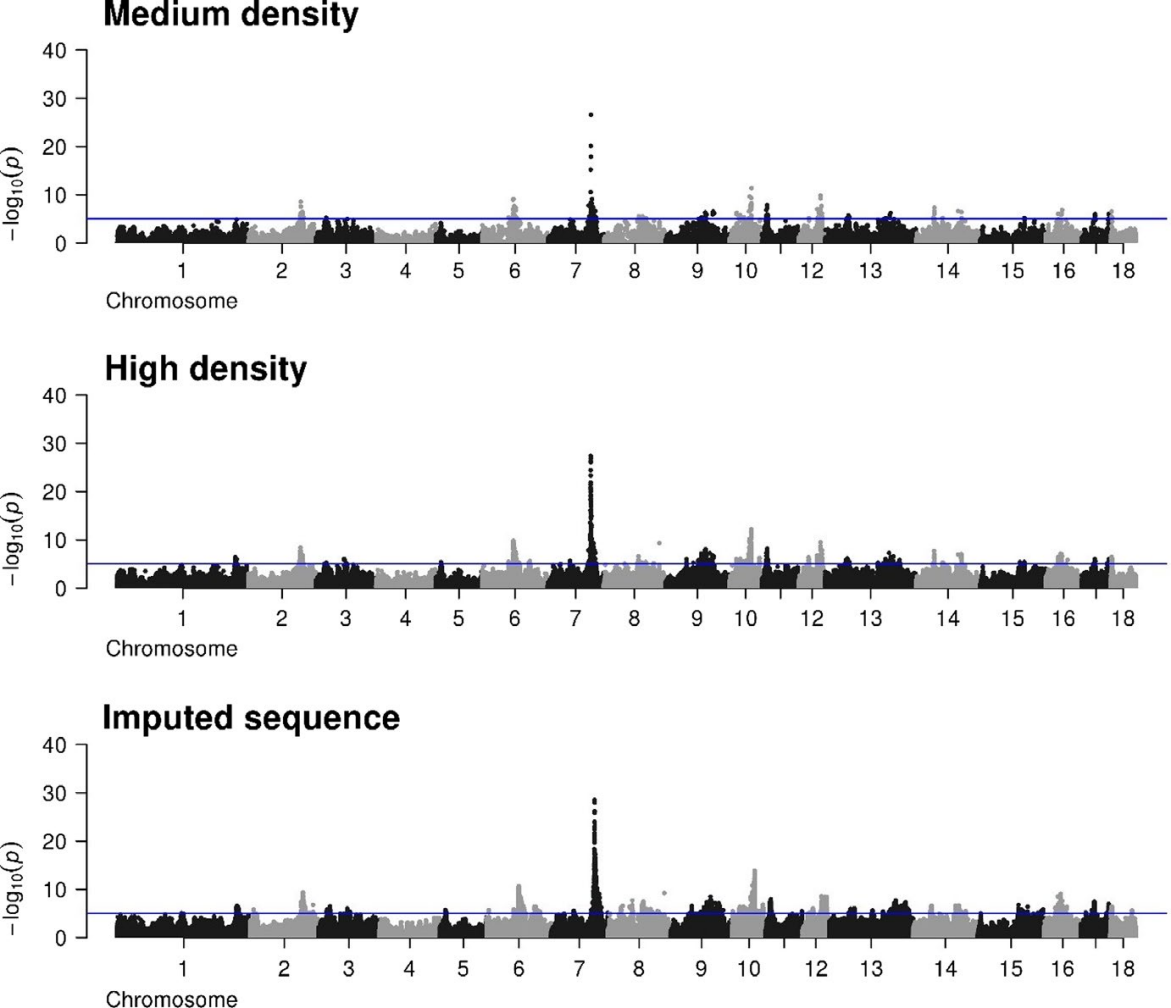
Manhattan plots for the number of teats using either medium‐density, high‐density or iWGS genotypes

In comparison with lower densities, more peaks are observed with iWGS (Figure 1). These peaks become also higher and more pronounced. The strongest peak found with all densities was located at approximately 103.4 MB on chromosome 7 and had a maximum –log10 (*p*‐value) of 28.5 using iWGS. Furthermore, highly significant peaks were found with all densities at chromosomes 2, 6, 10 and 12.

### 3.2 Genomic control

**Figure 2:**
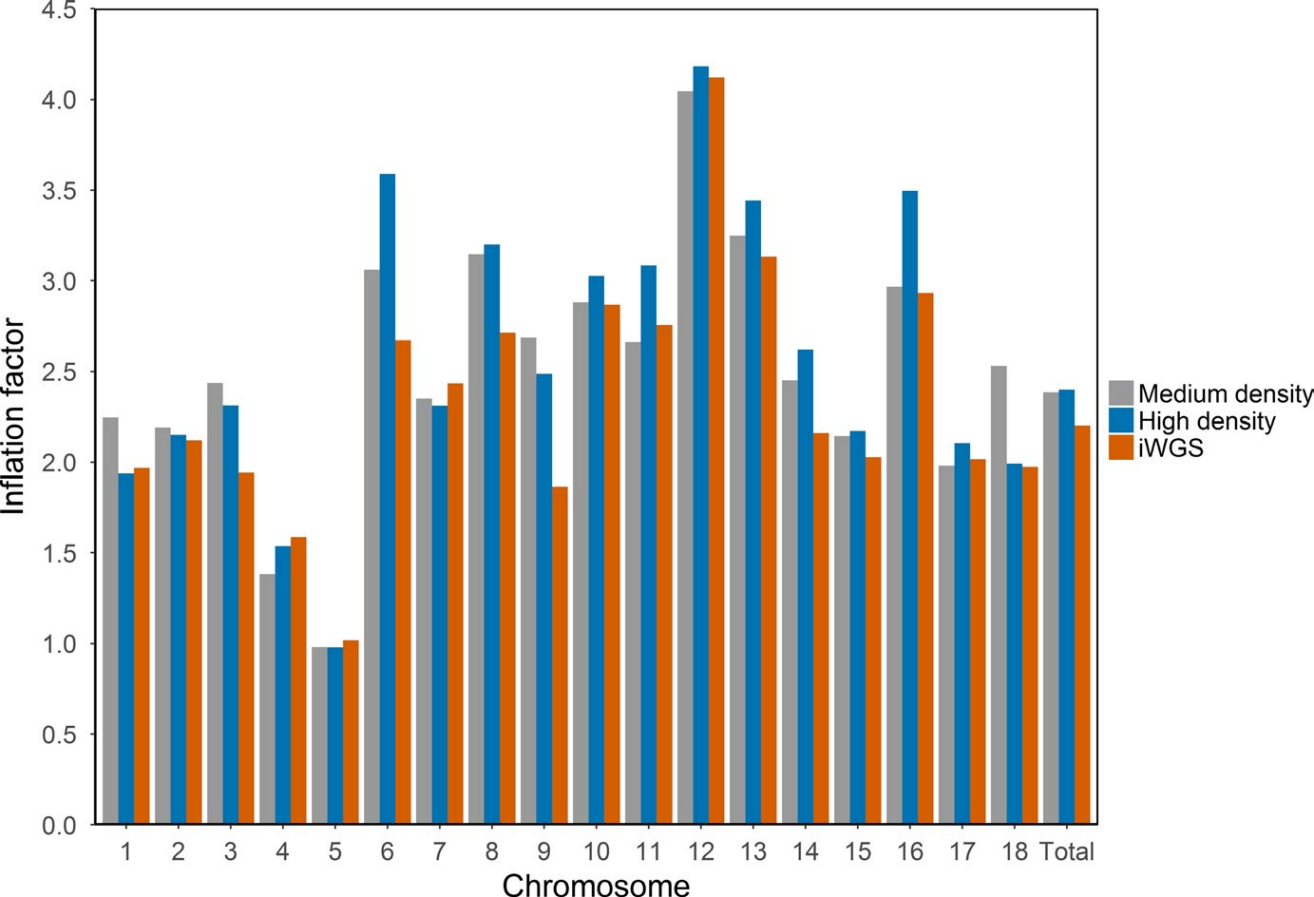
Inflation factors per chromosome and the total genome found with medium density, high density and iWGS

The genomic inflation factors at a whole‐genome level were all higher than 2 (average about 2.50) and were similar for the medium density, high densities, and iWGS. At the chromosome level, the genomic inflation factors also remained constant across the different densities (Figure 2), although genomic inflation factors varied between chromosomes, from 0.98 to 4.18 (Figure 2). Using a threshold of –log10 (*p*‐value) > 5 for iWGS, 67,784 SNPs were considered as significant without genomic control, 2,154 with genome‐wide control and 1,362 with chromosome‐specific genomic control. To investigate potential causes for these high genomic inflation factors, first the average genomic inflation factors over the 1,000 permutations were calculated for the chromosomes 4, 7 and 10. Values were around 1 and remained constant across chromosomes and densities (Table 1). Thus, when the association between genotype and phenotype was broken down with permutation testing, the genomic inflation factors decreased to 1.

**Table 1:** Significance thresholds and genomic inflation factors from permutation testing of chromosomes 4, 7 and 10 for medium and high densities and iWGS

Chromosome	Density	Threshold^a^	Genomic inflation factor^b^
4	Medium	4.178	0.997 (0.198)
	High	4.927	1.004 (0.204)
	iWGS	5.469	0.991 (0.196)
7	Medium	4.232	1.003 (0.207)
	High	4.922	1.003 (0.201)
	iWGS	5.449	1.005 (0.210)
10	Medium	4.100	1.003 (0.194)
	High	4.743	0.988 (0.188)
	iWGS	5.426	0.988 (0.185)

a *p*‐value thresholds are expressed as –log10 (*p*‐values).

b Averages and standard deviation within brackets over 1,000 permutations.

**Figure 3:**
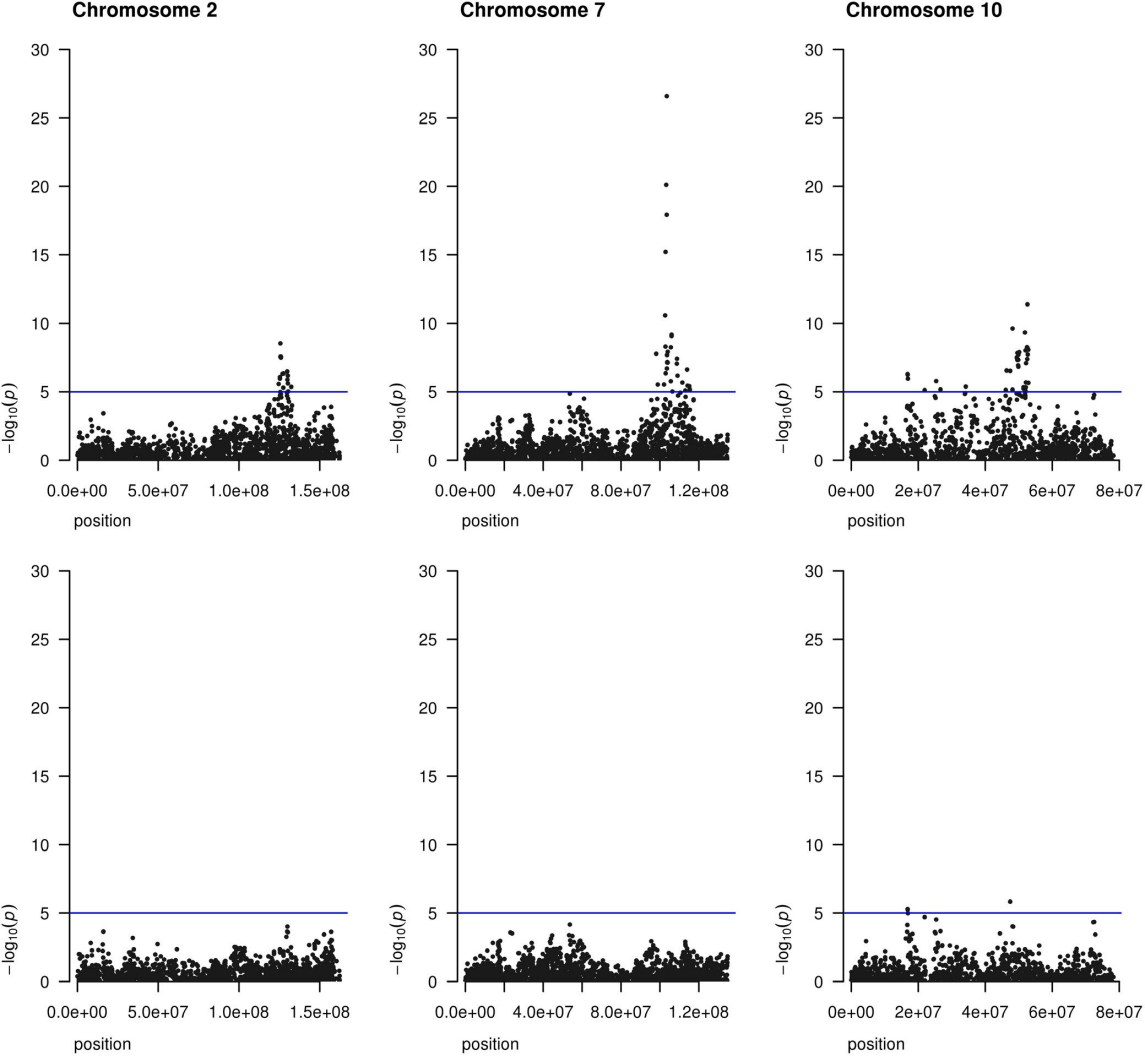
Manhattan plot of GWAS without (upper) or with 3 QTL as fixed effect (lower)

**Figure 4:**
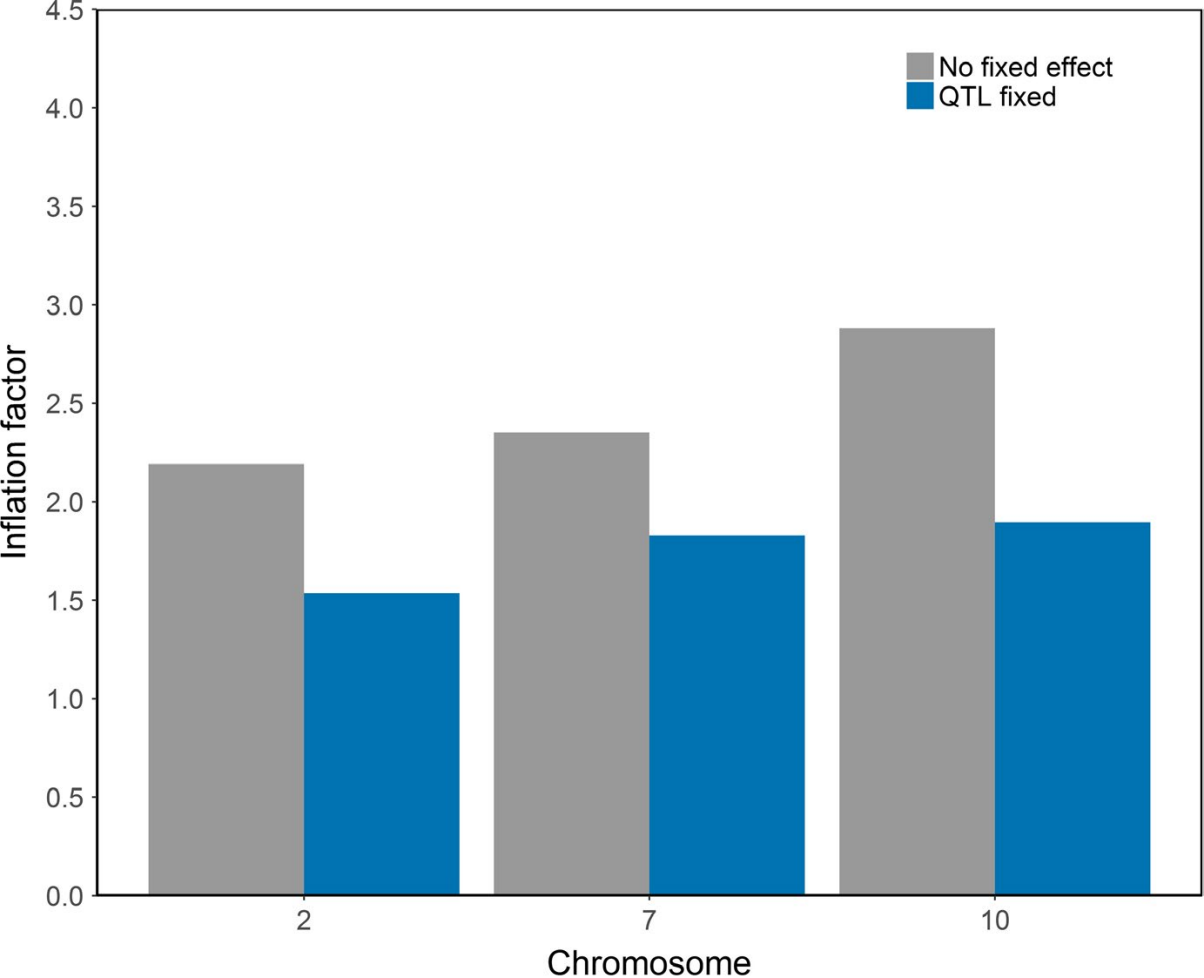
Genomic inflation factors of GWAS without (grey) or with 3 QTL as fixed effect (Blue)

**Figure 5:**
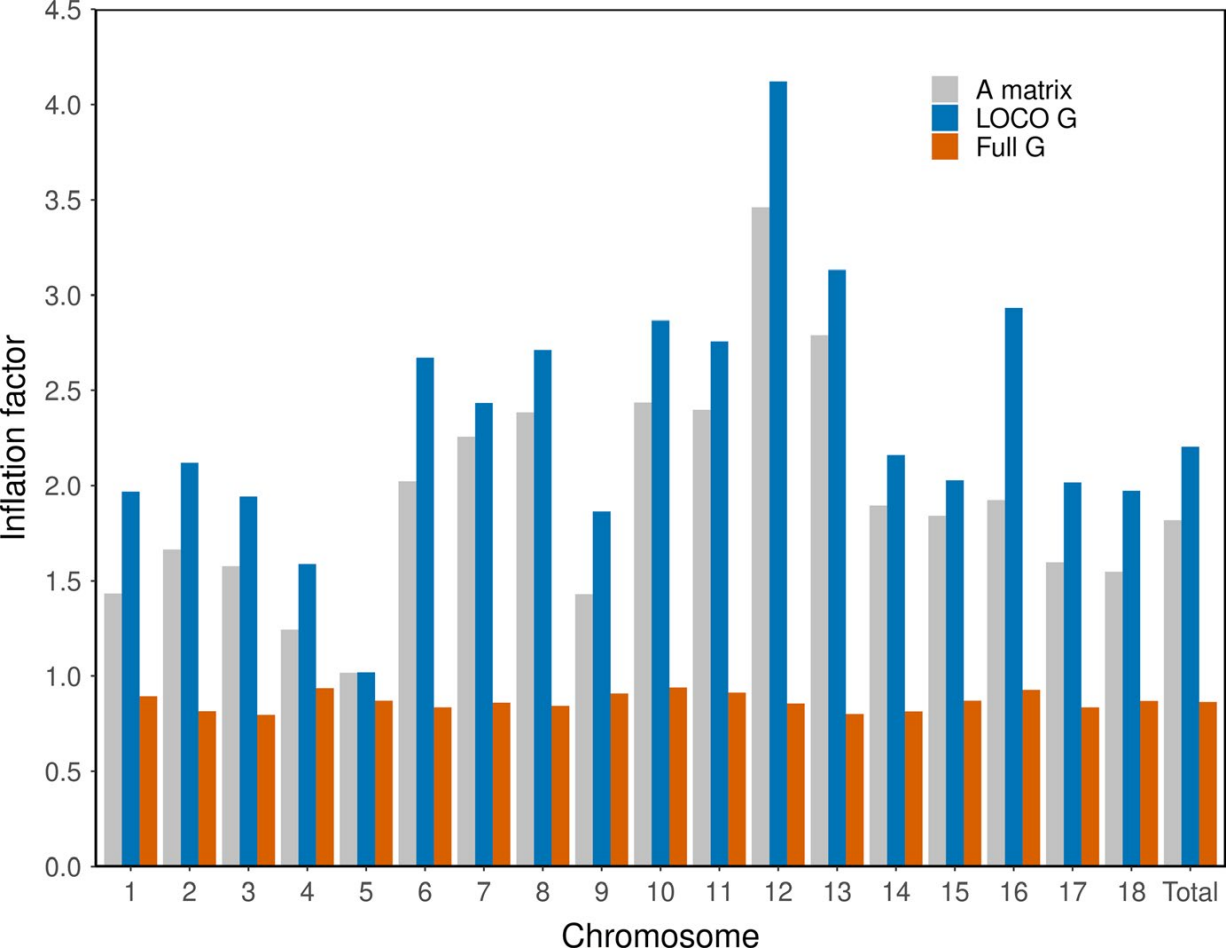
Inflation factors found per chromosome and across the whole genome using a pedigree relationship matrix (A matrix), a genomic relationship matrix based on the leave‐one‐chromosome‐out approach (LOCO G) and a genomic relationship matrix based on all iWGS markers (Full G)

Second, we also fitted three highly significant SNP (Table 2) on chromosome 2, 7 and 10 as fixed effect in the GWAS with medium‐density SNPs. By fitting the highly significant SNP as fixed effect, the peaks in the Manhattan plot disappeared (Figure 3), indicating that the association was removed. Although the genomic inflation factor dropped with at least 22% compared to the model without the most significant SNP as fixed effect (Figure 4), the genomic inflation factors were still not close to unity (that is, all above 1.5). Finally, the analysis was re‐run with the same phenotype and iWGS data sets but using a pedigree relationship matrix or a genomic relationship matrix based on all iWGS markers. Using the pedigree relationship matrix resulted in genomic inflation factors close to those obtained with the LOCO approach (Figure 5). However, using a genomic relationship matrix based on all iWGS markers resulted in all genomic inflation factors per chromosome close to 1 (Figure 5).

**Table 2:** Details on the top 4 QTL used as a fixed effect in the GWAS model

Chromosome	Position^a^	−log10(*p*‐value)	SNP effect
2	125.63	9.1	−0.13
7	103.5	26.6	0.34
10	525.9	11.5	−0.15

a Position is given in mega base pairs (MB).

### 3.3 Significance thresholds

Permutation testing was used to set the baseline value for the significance threshold for –log10 (*p*‐value). The significance thresholds found with permutation testing for the medium‐density SNP chip were 4.1 and increased to 5.5 when the marker density increased to iWGS. In contrast to the genomic inflation factors, significance thresholds were constant across chromosomes (Table 1).

The level and behaviour of the significance threshold were dependent on the number of independent tests applied to the Bonferroni correction. When the total number of SNPs was used as the number of independent tests, the threshold increased from 5.8 to 8.3 when moving from medium density to iWGS (Table 3). However, the significance thresholds remained constant at about 3.6 when the total number of independent chromosome fragments was used as the number of independent tests (Table 3), because the total numbers of independent chromosome fragments were also constant at approximately 200 across densities.

**Table 3:** Significance thresholds of a Bonferroni correction using the total number of SNPs or the number of independent chromosome fragments for medium and high densities and iWGS

	Bonferroni_total^a^		Bonferroni_M*_e_* b		FDR_BH^c^	FDR_BY^d^
	# SNP	Threshold^e^	M_e_ f	Threshold^e^	Threshold^e^	Threshold^e^
Medium density	34,588	5.84	198.9	3.60	2.48	4.19
High density	491,169	6.99	193.4	3.59	2.44	4.38
iWGS	10.2 M	8.31	223.4	3.65	2.57	4.54

a Bonferroni_total = 0.05/total number of SNPs.

b Bonferroni_*M*_e_ = 0.05/ *M*_e._

c The false discovery rate (FDR) computed following Benjamini and Hochberg (1995) (BH).

d The false discovery rate (FDR) computed following Benjamini and Yekutieli (2001) (BY).

e Significance thresholds are expressed as –log10 (*p*‐values).

f *M*_e_ is the number of independent chromosome fragment calculated with the formula proposed by Goddard et al. (2011).

Using the FDR, the significance threshold remained more constant, at about 2.50 across the different SNP densities when using the BH procedure, while a slight increase was observed, from 4.19 to 4.54, with increasing marker density when using the BY procedure.

**Figure 6:**
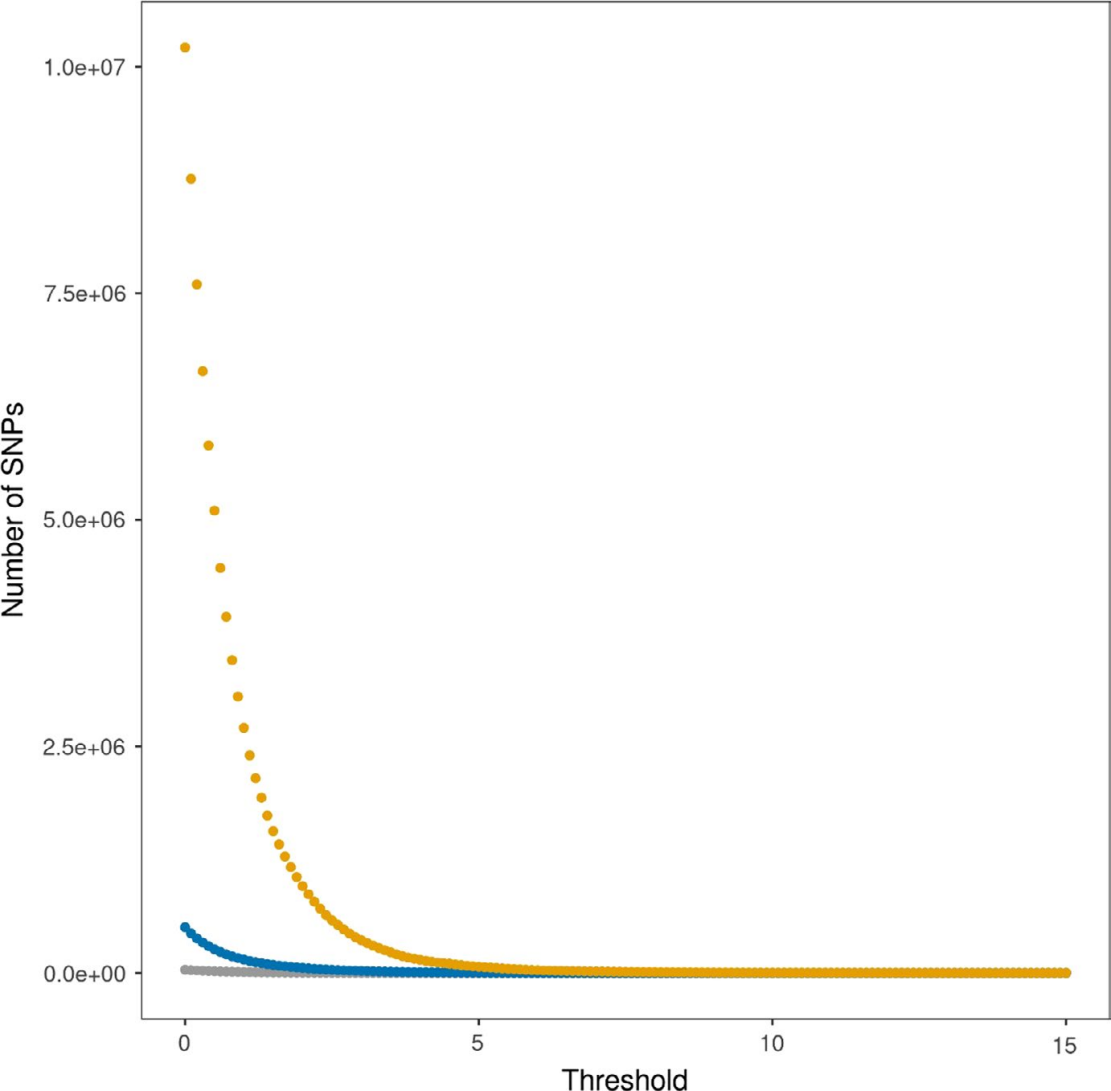
Number of SNPs with a significance level above a range of significance thresholds (without correction) for medium density (grey), high density (blue), and iWGS (orange)

**Figure 7:**
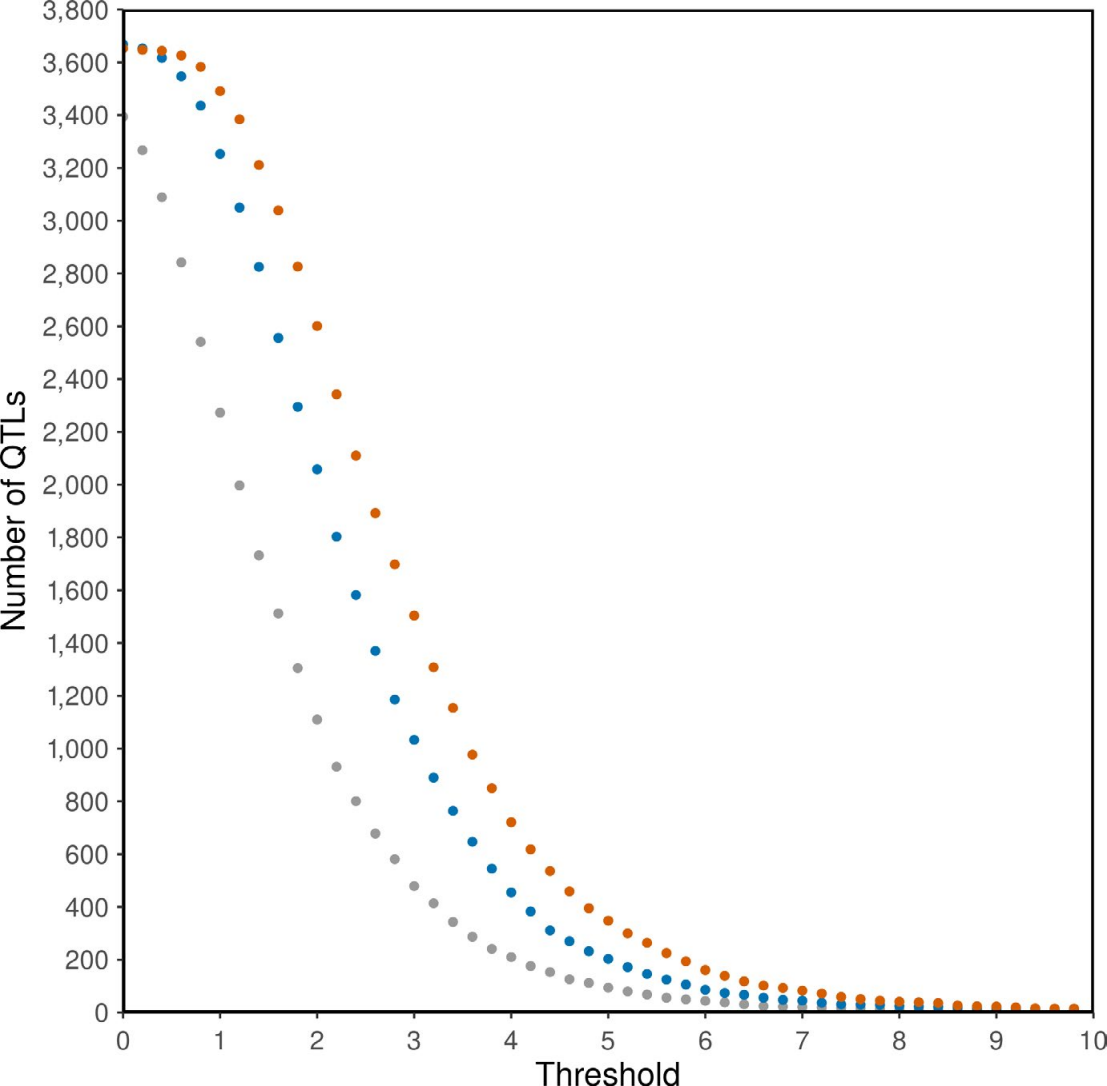
Number of QTL regions with a significance level above a range of significance thresholds (without correction) for medium density (grey), high density (blue) and iWGS (orange)

The effect of significance threshold on the number of SNPs above a significance threshold is illustrated in Figure 6, and there was an obvious effect of genotype density on the number of identified significant SNPs. Figure 7 shows the number of QTL regions (a region includes all SNPs within a 0.5 Mb region to the left and right of the most significant SNP) is also highly dependent on genotype density and significance threshold. For example, for iWGS, the number of QTL regions was equal to 36 with a significance threshold of 8.3 (that is, with the Bonferroni correction based on the total number of SNPs), to 264 with a signification threshold of 5.4 (that is, a threshold obtained from the permutation test) and to 977 with a significance threshold of 3.6 (that is, with the Bonferroni correction based on the number of independent chromosome fragments).

## 4 DISCUSSION

The objective of this study was to evaluate and compare different statistical testing procedures in order to minimize the chance of finding false positives for a GWAS using data from a commercial pig line.

**Figure 8:**
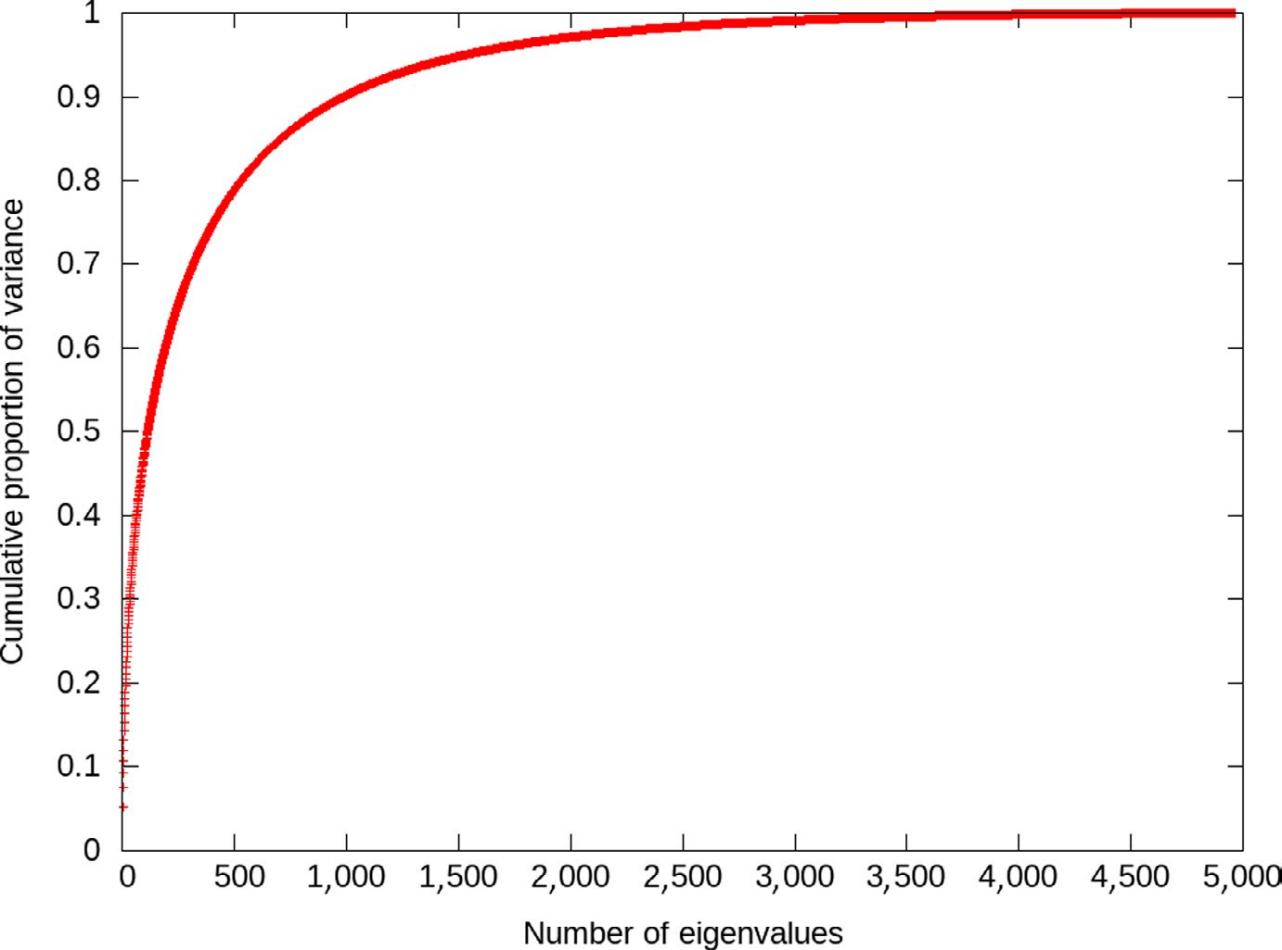
Cumulative proportion of variance explained in the genomic relationship matrix by its eigenvalues

Genomic inflation factors in this study were high (that is, all above 2), suggesting that we need to adjust for population stratification in the data. Accounting appropriately for population stratification is important to avoid strong genomic inflation and consequently avoid false positives (Cardon & Palmer, 2003; Hinrichs et al., 2009; Patterson, Price, & Reich, 2006; Price et al., 2006). In other GWAS using commercial pig lines, the genomic inflation factors were much lower (Diniz et al., 2014; Lopes, Bastiaansen, Harlizius, Knol, & Bovenhuis, 2014; Luo et al., 2012; Sanchez et al., 2014). For example, Lopes et al. (2014) found genomic inflation factors of 1.13 for the number of teats using 60K Large White genotypes. The difference between literature and this study was likely not due to a different density of SNPs since the genomic inflation factors did not vary between the densities used in this study. Instead, we showed that the LOCO approach in GCTA is a likely reason for the genomic inflation in our population. With the LOCO approach, the genomic relationship matrix used to account for population stratification only included the other chromosomes than the one where the tested SNP is located. It has been shown that the LOCO approach improves the power of GWAS in human studies (Lippert et al., 2011; Listgarten et al., 2012; Yang et al., 2014) because the tested SNP is not double fitted in the model. However, in human studies, most individuals are often unrelated, while in livestock breeding populations, many strong family relationships exist. For example, in this data set, each individual had on average 2.2 full sibs and 42 half‐sibs. This complicated family structure is also illustrated by the eigenvalues of the genomic relationship matrix computed using the medium‐density genotypes (Figure 8). It can be observed that a few eigenvalues explain a relatively large proportion of the variance (e.g., the 10 first largest eigenvalues explain >17% of the variance). Therefore, our results confirm that the LOCO approach does not account appropriately for the population stratification in a pig breeding population and probably also in many other livestock populations used for a GWAS. Furthermore, we showed that the pedigree relationship matrix does not account appropriately for the population stratification neither. Hence, fitting a genomic relationship matrix using all chromosomes might be more appropriate than the other options and might diminish the need to adjust for population structure.

Although the genomic inflation factors did not differ a lot between the different SNP densities, genomic inflation factors varied largely across the chromosomes. A likely explanation might be that there was a different level of association between the SNP on the chromosome and the trait of interest. Strong association between SNP and the trait of interest can cause inflation of the test statistic in the neighbouring regions because of LD between these SNPs, and many more SNPs were significant when an average genomic control was performed across the chromosome. Therefore, we suggest that genomic control should be applied per chromosome.

### 4.1 Definition of the significance threshold

Permutation testing is a method to derive empirical thresholds that can be used to validate thresholds found with less computationally intensive approaches, such as the Bonferroni correction or the FDR. The procedure for permutation testing used in this study assumed that the observations are independent (Churchill & Doerge, 1994). Because the 900 principal components suggest that there are many families (Patterson et al., 2006), it is likely that with 1,000 permutations, the dependency between observations between family members is broken down and the assumption of the independence between observations is fulfilled.

The empirical thresholds established by permutation testing were used as a reference to validate the thresholds established by the less computationally intensive Bonferroni correction and FDR. Based on the empirical thresholds, it was expected that the significance threshold increased with increasing SNP density. However, the significance threshold based on the Bonferroni correction using the total number of SNPs was much higher than the empirical thresholds; that is, the threshold based on the Bonferroni correction increased from 5.84 to 8.31, whereas the empirical thresholds increased from 4.1 to 5.5. The assumption that the total number of SNPs is equal to the number of independent tests is too conservative, since there is LD between the SNPs.

**Figure 9:**
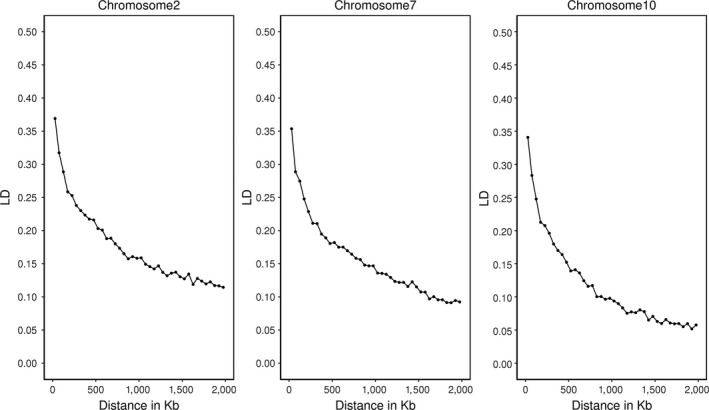
LD decay on chromosome 2, 7 and 10 between medium‐density SNPs. LD was measured as *r* ^2^ between SNP in bins of 50 kilo‐base pair (KB)

The Bonferroni correction with the number of independent chromosome fragments did account for an average LD between SNPs across the genome. However, even though using the number of independent chromosome fragments to represent the LD structure of the data better, the significance thresholds did not increase with increasing SNP density and the thresholds were quite low compared to those ones established with permutation testing, for example, 5.4 with permutation testing and 3.4 with the Bonferroni correction for iWGS. Dudbridge and Gusnanto (Dudbridge & Gusnanto, 2008) also reported underestimated significance thresholds when the number of independent chromosome fragments was used with the Bonferroni correction. They argued that using principal component analysis to estimate the number of independent chromosome fragments might not be an appropriate method, and therefore, the thresholds were lower than expected. In this study, the number of independent chromosome fragments was defined as the inverse of the variance of the off‐diagonal elements of the genomic relationship matrix. However, it could be argued that this method might be not appropriate because it assumed uniformity of LD patterns across the genome (Goddard 2009). This assumption is not valid because LD patterns differ across chromosomes (Figure 9). Ignoring the variation in LD patterns could lead to an underestimation of the number of independent chromosome fragments and subsequently to underestimated thresholds, especially with iWGS.

### 4.2 Applications

The aim of this study was to investigate different approaches to test for significance in a GWAS with iWGS. The number of false positives can be minimized by a combination of applying genomic control and then choosing an appropriate threshold. We expected the combination of the genomic control and the FDR to result in an overestimation of the significance thresholds. Additional analysis confirmed that the combination of FDR and genomic control inflates the significance threshold, because the FDR threshold was 4.54 without considering genomic control (Table 3), and increased to 7.64 with considering first genomic control (result not shown). We hypothesize the high thresholds were caused by an entanglement of theory behind genomic control and FDR. Genomic control corrects *p*‐values for their deviations from the expected distribution. Also, the FDR establishes a new distribution by applying a cut‐off for *p*‐values that do not follow the expected distribution under the null hypothesis. So, both genomic control and FDR rescale the expected distribution and thus they are confounded in their underlying procedures.

Fitting the full genomic relationship matrix based on all chromosomes might alleviate the need for genomic control. When the aim is to find QTL regions that are associated with a trait of interest, we recommend to use the FDR following the BY approach to establish a significance threshold that is adjusted multiple testing. When the aim is to pinpoint a specific mutation, the more conservative Bonferroni correction based on the total number of SNPs appears more appropriate. However, it is still not satisfying that adjusting for the total number of SNPs is needed to account for increasing number of tests with iWGS, and a more appropriate method to determine the number of independent test is required.

## 5 CONCLUSION

The objective of this study was to compare different significance testing procedures and to establish guidelines for significance testing in a GWAS using either medium‐density genotypes, high‐density genotypes or iWGS for a commercial pig population. We found that genomic inflation factors did not differ between different densities but did differ between chromosomes. In addition, a genomic relationship matrix based on the leave‐one‐chromosome‐out approach does not account appropriately for population stratification and gave strong genomic inflation in this pig breeding population. Based on our results, we recommend to use either genomic control in combination with Bonferroni correction (using the total number of SNPs and depending on the aim of the study, relax the significance level) or the FDR without applying genomic control. Fitting the full GRM based on all chromosomes might alleviate the need for genomic control.

## CONFLICT OF INTEREST

The authors declare that there is no conflict of interest regarding the publication of this article.

6

## Data Availability

The data that support the findings of this study are from Topigs Norsvin. Restrictions apply to the availability of these data, which were used under licence for this study. Data are available from the authors with the permission of Topigs Norsvin.
